# Care from the Cultural Perspective in Women with Physiological Pregnancy: a Meta-Ethnography

**DOI:** 10.17533/udea.iee.v37n1e03

**Published:** 2019-01-06

**Authors:** Iliana Milena Ulloa Sabogal, Lucy Muñoz de Rodríguez

**Affiliations:** 1 Nurse, Masters. Professor, Universidad Industrial de Santander, Bucaramanga, Colombia. Email: imulloa@uis.edu.co Universidad Industrial de Santander Universidad Industrial de Santander Colombia imulloa@uis.edu.co; 2 Nurse, Masters in Nursing with Emphasis on Family Health. Professor Emeritus, Universidad Nacional de Colombia. Bogotá, Colombia. Email: lmunozdr@unal.edu.co Universidad Nacional de Colombia Universidad Nacional de Colombia Colombia lmunozdr@unal.edu.co

**Keywords:** culture, pregnancy, transcultural nursing, qualitative research, review literature as topic., cultura, embarazo, enfermería transcultural, investigación cualitativa, literatura de revisión como asunto., cultura, gravidez, enfermagem transcultural, pesquisa qualitativa, literatura de revisão como assunto.

## Abstract

**Objective.:**

This work sought to conduct an interpretative synthesis of qualitative studies on the phenomenon of care from the cultural perspective in women with physiological pregnancy.

**Methods.:**

The Meta-ethnography method was used with the seven traditional phases by Noblit and Hare to describe the knowledge derived from the results of qualitative studies with relation to the study phenomenon. A bibliographic search was carried out in seven databases. Twenty-nine qualitative studies were pre-selected of which 23 complied with the quality criteria of the Critical Appraisal Skills Program.

**Results.:**

Upon synthesizing the studies selected, 12 thematic categories emerged: pregnancy: a natural phenomenon in the woman’s life; spirituality and family support; the midwife; positive and negative feelings; physical exercise; comfort and rest; feeding; avoid consumption of non-beneficial substances; intrauterine stimulation; heat and cold; sexuality during pregnancy; and traditional beliefs and myths.

**Conclusion.:**

Synthesis of the studies permitted developing a line of argument, which reveals that the care practices of pregnant women have a cultural legacy of beliefs, values, myths, and customs that are aimed at guaranteeing the protection of the mother and of her unborn child.

## Resumen

**Objetivo.:**

Realizar una síntesis interpretativa de los estudios cualitativos sobre el fenómeno del cuidado desde la perspectiva cultural en mujeres con embarazo fisiológico.

**Métodos.:**

Se empleó el método Metaetnográfico con las siete fases tradicionales de Noblit y Hare, para describir el conocimiento derivado de los resultados de estudios cualitativos con relación al fenómeno de estudio. Se realizó una búsqueda bibliográfica en siete bases de datos. Se preseleccionaron 29 estudios cualitativos, de los cuales 23 cumplieron con los criterios de calidad del *Critical Appraisal Skills Programme*.

**Resultados.:**

Al sintetizar los estudios seleccionados, surgieron doce categorías temáticas: El embarazo: fenómeno natural en la vida de la mujer, la espiritualidad y el apoyo familiar, la comadrona, sentimientos positivos y negativos, el ejercicio físico, la comodidad y el descanso, la alimentación, evitar el consumo de sustancias no beneficiosas, la estimulación intrauterina, el calor y frío, la sexualidad en el embarazo y, las creencias tradicionales y los mitos.

**Conclusión.:**

La síntesis de los estudios permitió el desarrollo de una línea de argumentación la cual revela que las prácticas de cuidado de la mujer gestante cuentan con un legado cultural de creencias, valores, mitos y costumbres que están orientadas a garantizar la protección de la madre y del niño por nacer.

## Resumo

**Objetivo.:**

Realizar uma síntese interpretativa de estudos qualitativos sobre o fenômeno do cuidado a partir da perspectiva cultural em mulheres com gestação fisiológica.

**Métodos.:**

O método Meta-etnografia foi utilizado com as sete fases tradicionais de Noblit e Hare, para descrever o conhecimento derivado a partir dos resultados de estudos qualitativos em relação ao fenômeno de estudo. Uma busca bibliográfica foi realizada em sete bases de dados. Vinte e nove estudos qualitativos foram pré-selecionados, dos quais 23 preencheram os critérios de qualidade do Critical Appraisal Skills Program.

**Resultados.:**

Ao sintetizar os estudos selecionados, emergiram doze categorias temáticas: Gravidez: um fenômeno natural na vida das mulheres; espiritualidade e apoio familiar; a parteira; sentimentos positivos e negativos; exercício físico; conforto e descanso; a alimentação; evitar o consumo de substâncias não benéficas; estimulação intra-uterina; o calor e o frio; sexualidade na gravidez; e, crenças e mitos tradicionais.

**Conclusão.:**

A síntese dos estudos permitiu o desenvolvimento de uma linha de argumentação, o que revela que as práticas de cuidados das mulheres grávidas, têm um legado cultural de crenças, valores, mitos e costumes que são destinadas a assegurar a proteção da mãe e seu feto.

## Introduction

Pregnancy is a life experience and one of the most important events within the vital cycle of the woman and the family during which women develop behaviors and perform care practices for themselves and the unborn child to maintain health, care for their diseases, and conserve their wellbeing and that of their child, according to Muñoz.(1) The behaviors and care practices women have during the prenatal stage depend on the social structure and on the ethno-historic and environmental context, that is, on the culture in which they grow and live.(2,3) Leininger states that culture was the broadest, most comprehensive, holistic and universal aspect of human beings, and that caring for people should be carried out from a transcultural vision. In this sense, Leininger conceives cultural care as the dual, central, and dominant construct within the theory of Culture Care Diversity and Universality,(4) the theorist refers to *cultural care* as: “The values, beliefs, and structured and known expressions of a cognitive form that aid, support, facilitate, or train people or groups to maintain their health or wellbeing, improve their situation or way of life, prevent disease or confront disabilities or death”.(5) From this perspective, this proposal constitutes a humanistic, scientific, and comprehensive alternative in caring for pregnant women, recognizing that it is not exempt from the cultural constructions, where its values, customs and beliefs have direct influence on the care practices and each culture defines them from their particular vision of life to be transmitted from generation to generation and, thus, be perpetuated over time.(6) 

Nursing and other disciplines have studied the care of pregnant women from the meanings and experiences to describe, discover, or explore the practices of cultural care conducted by women during the prenatal stage. However, no research was found that have integrated, synthesized, analyzed, and interpreted the results of primary qualitative studies, upon which emerged the interest of carrying out a Meta-Ethnography on the theme. Knowledge derived from this work will be essential to further understand the care of pregnant women and their families from a transcultural perspective. Likewise, interpretation of caring for pregnant women, from this vision, will support the application of the strategic framework of the Policy of Comprehensive Health Care in Colombia, which recognizes the health problems are generated or enhanced by environmental, social, and cultural conditions,(7) which must be considered within the guidelines of the Comprehensive Care Route in Maternal Perinatal Health.(8) The objective of this study was to conduct an interpretative synthesis of the qualitative studies on the phenomenon of care from the cultural perspective in women with physiological pregnancy. 

## Methods

The methodology used was the meta-ethnography developed by Noblit and Hare,(9) which permits conducting a combination of results in interpretative manner rather than aggregative, to generate a higher level of analysis that contributes much more than the individual findings of each investigation.The seven traditional phases by Noblit and Hare were followed for the meta-ethnography which overlap and repeat as the synthesis advances; these include:

### Phase I: Start of the process.

Interest was established in conducting an interpretative and explicative synthesis of cultural care in women with physiological pregnancy.

### Phase II: Decide what is relevant for the initial interest.

The meta-ethnography included original articles and research works from the Masters in Nursing or other disciplines, which described care from the cultural perspective in women with physiological pregnancy, published in full text, in English, Portuguese, and Spanish between 2000 and 2016. The search strategy used MeSH and DeSH terms “care/cuidado”, “culture/cultura”, “cultural care/cuidado cultural”, “care practices/prácticas de cuidado”, “culturally competent care/cuidado culturalmente competente”, “beliefs/creencias”, “pregnancy/embarazo” y “ethnography/etnografía”. The studies were recovered through a search in PubMed, Lilacs, Scielo, Ovid, Academic Search Complete, Medline Complete, ScienceDirect databases and the Repository at Universidad Nacional in Colombia. Finally, the studies selected were evaluated with the quality criteria from the Critical Appraisal Skills Program (CASP) to assess qualitative studies, (10) which are considered fulfilled if there is internal validity of the study, rigorous analysis of the data, and external validity of the findings. 

### Phase III: Reading of the studies.

This phase included the reading and rereading of the studies, which permitted extracting the results and conclusions from each of the studies the key metaphors. 

### Phase IV: Determine how the studies are related.

A list was made of the key metaphors extracted and these were organized to facilitate their comparison, within and between studies.

### Phase V: Transfer the studies one within another.

This phase applied the reciprocal translation process among the studies, which consisted in examining the list of the key metaphors in relation to other metaphors within each study. At the end of this phase, it was established that the studies were directly comparable, that is, the key metaphors extracted expressed similarities among the findings of each study.

### Phase VI: Synthesize the translations.

The key metaphors were grouped into 12 thematic categories that represented the characteristics or dimensions of cultural care in women with physiological pregnancy. This phase implied new re-readings of the original studies to re-conceptualize the results, that is, the generation of a new interpretation from a second level of analysis.

### Phase VII: Express the synthesis in a final product.

This phase consisted in analyzing the interpretations obtained in the synthesis of translations; this again implied reading the studies and the comparison of the 12 thematic categories, which gave way to what Noblit and Hare describe as "line of argument”, which is understood as the construction of a reinterpretation of the findings of the studies. Generation of the line of argument permitted creating a new interpretative synthesis of care from the cultural perspective in women with physiological pregnancy.

## Results

**Figure 1:**
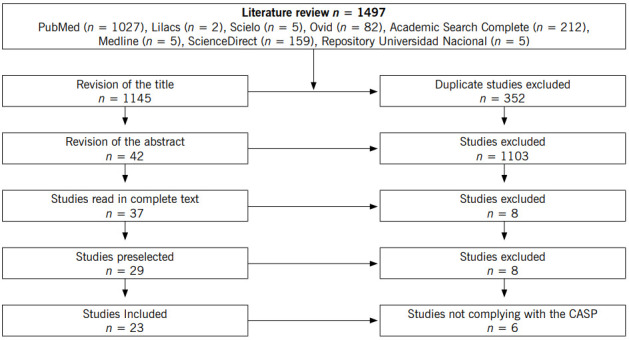
Article selection process

The selection process of the articles to be analyzed identified 1497 bibliographic sources from which 21 articles were obtained and two Masters Theses in Nursing were finally included in the Meta-Ethnography, as shown in Figure 1.

Table 1 shows the objective, form of collecting the information, and methodological design of the studies included in this Meta-ethnography.

**Table 1:** Characteristics of the 23 qualitative studies included in the Meta-ethnography

Authors/Year	Objetive	Country	Participants	Collection of information	Methodological Desing
Ahlqvist M, Wirfäl E. (2000) (11)	To explore the cultural beliefs on feeding and health during pregnancy and breastfeeding.	Sweden	14 women	Open and focal interviews	Grounded theory
Flores C. (2003) (12)	To describe and analyze the health representa- tions and practices of the process of preg- nancy, delivery, puerperium in the daily lives of Zapoteca women from Yahuío, Sierra Norte de Oaxaca.	Mexico	42 women	Semistructured interviews and in- depth interviews	Ethnographic study
Medina A, Mayca J. (2006) (13)	To understand and revise the cultural aspects and customs impacting upon the processes of pregnancy, delivery, and puerperium.	Peru	40 participants among mid-wives, health promotors, and users	In-depth interviews, focal groups and participant observa- tion	Ethnographic, descriptive study
Giraldo DI. (2007) (3)	To discover the meaning of care pregnant women have of themselves and of their unborn child from their beliefs, values, and practices, from the transcultural perspective by M. Leini- nger, in the pre-delivery stage, from the El Pinar neighborhood in Medellín.	Colombia	12 pregnant women	Ethnographic inter- view by Spradley	Ethnographic study based on ethnonursing
Argote LA, Vásquez ML. (2007) (14)	To explore the care of themselves and of their unborn child in a group of displaced pregnant women, who live in the Pampas de Mirador neighborhood in Cali.	Colombia	9 pregnant women and 4 general informants	In-depth interviews and observation	Ethnographic study based on ethnonursing
Chávez R. et al., (2007) (15)	To know the traditional self-care of native women during pregnancy, delivery and the newborn.	Perú	5 mothers, 4 pregnant women, 3 puerperants and 4 midwives	Semistructured interviews	Ethnographic study
Bernal MC et al., (2007) (2)	To explore the meaning of caring for them- selves and of their unborn child for a group of displaced pregnant women residing in Bogotá, from their own beliefs and practices.	Colombia	12 pregnant women	Individual unstruc- tured in-depth in- terview, focal group and observation	Ethnographic study based on ethnonursing
Suárez DP, Muñoz de Rodríguez L. (2008) (16)	To discover the meaning of physical exercise during the prenatal stage from the beliefs and practices of pregnant women in the prenatal control program at the E.S.E Hospital San Rafael in Girardot.	Colombia	8 pregnant women	In-depth interviews and direct partici- pant observation	Ethnographic study based on ethnonursing
Hernández LM. (2008) (17)	To describe the meaning of caring for them- selves and for their unborn child, from their values, beliefs, and practices, for a group of pregnant women from the locality of Engativá.	Colombia	8 pregnant women	Semistructured in- depth interviews	Ethnographic study based on ethnonursing
Grewal S; Bha- gat R; Balnea- ves L. (2008) (18)	To describe the knowledge and cultural tradi- tions surrounding the perinatal experiences of Punjabi immigrant women and the ways the beliefs and traditional practices are legitimized and incorporated into the context of Canadian medical care.	Canada	15 women	Individual inter-views	Naturalist descrip- tive study
Rátiva N, Ruíz de Cárdenas CH. (2009) (19)	To describe the meaning of the care practices of pregnant adolescents and their unborn child attending prenatal control in the Candelaria Primary Care Unit (UPA, for the term in Span- ish) from Hospital Vista Hermosa of locality 19, Ciudad Bolívar from the beliefs, practices, and values between March and June 2007.	Colombia	Colombia 8 pregnant women	In-depth interview and observation	Ethnographic study based on ethnonursing
Ribeiro M, Ferreira S. (2010) (20)	To analyze the feeding practices during preg- nancy from the perspective of pregnant women and puerperants living in a complex of favelas in Rio de Janeiro, Brazil.	Brasil	18 pregnant women and 8 puerperants	Semistructured Interview	Study adopted the interpretative theory
Rodríguez I, Bernal MC (2010) (21)	To describe the meaning of caring for them- selves of a group of pregnant adolescents and their unborn child, related to feeding, from their practices, beliefs, and cultural values, who at- tended prenatal control in the Primary Care Unit (UPA) at Candelaria la Nueva, Hospital Vista Hermosa, Ciudad Bolívar, Locality 19 of Bogotá, in 2007.	Colombia	8 pregnant women	Unstructured, in- depth ethnographic interview	Ethnographic study
Guarnizo M, Pardo MP. (2011) (22)	To describe the meaning of sexuality for preg- nant women.	Colombia	9 pregnant women	Semistructured interviews	Ethnographic study
Ramos CP, Muñoz de Rodríguez L. (2011) (23)	To describe the cultural practices of caring for indigenous pregnant women who live in the Zenú reservation in the Córdoba Sabana.	Colombia	10 indigenous pregnant women	Observation and in- depth interview	Ethnographic study based on ethnonursing
Barragan D, et al., (2011) (24)	To evaluate the integration of the practices of cultural health and Western medicine during pregnancy in women of Mexican origin from different levels of acculturation.	The United States	15 women	Semistructured interviews	Qualitative study
Choudhury N, Ahmed SM. (2011) (25)	To explore existing maternal care practices dur- ing pregnancy, delivery, and post-delivery period of women from extremely poor homes included in the CFPR II program (2007-2012).	Bangladesh	12 nursing mothers and 8 pregnant women	In-depth interviews	Exploratory study
Wulandari LPL, Whelan AK (2011) (26)	To explore the beliefs, attitudes, and behaviors of pregnant women in Bali, Indonesia.	Indonesia	18 pregnant women	In-depth interviews	Descriptive study
Agus Y, Horiuchi S, Porter SE. (2012) (27)	To describe the perception of women on themes related with traditional beliefs during their preg- nancy in the rural area of Indonesia.	Indonesia	16 women	Focal groups using semistructured interview	Cross-sectional exploratory study
Rendón BJ, Ruíz de Cárde- nas CH. (2012) (28)	To describe the meaning of the cultural care practices of pregnant women with themselves and their unborn children in prenatal control, in the San Antonio hospital in the municipality of Villamaría, Caldas, from February to August 2011.	Colombia	10 pregnant women	In-depth interview, participant observa- tion, and field notes	Ethnographic study based on ethnonursing
De- Graft Aikins A. (2014) (29)	To explore the feeding beliefs and practices of women in Ghana during pregnancy.	Ghana	35 women	Interviews	Exploratory study
Higginbottom GM, et al., (2014) (30)	To understand the feeding practices and eth- nocultural health and how these are integrated into a particular social context of cultural adaptation.	Canada	10 women	Semistructured interview	Case study that incorporates a participative ap- proach
Muñoz M; Pardo MP. (2016) (31)	To describe the meaning of cultural care prac- tices in a group of pregnant adolescents attend- ing prenatal control in the Niño Jesús Hospital in Barranquilla, Colombia.	Colombia	10 pregnant women and 12 nurses	Ethnographic interviews	Ethnographic study based on ethnonursing

**Figure 2:**
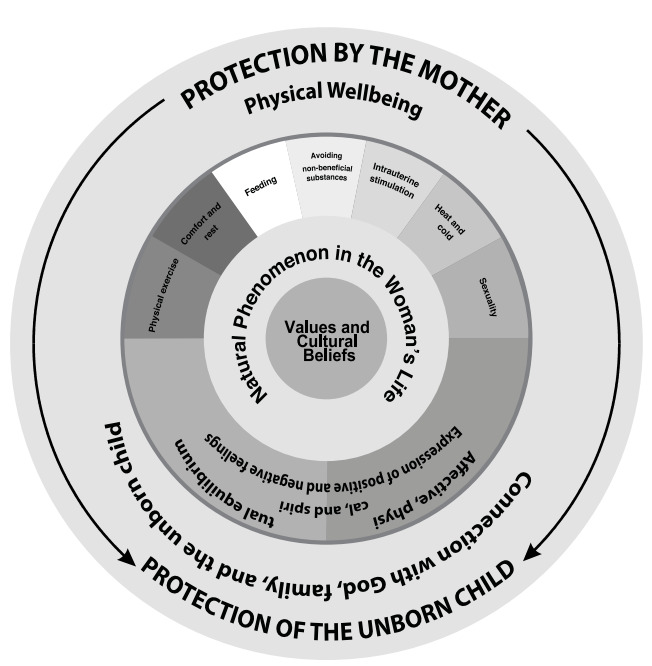
Line of argument: “care from the cultural perspective aimed at the protection of the mother and her unborn child”

The synthesis of translations among studies permitted identifying 12 thematic categories, which linked together allowed creating a new interpretative synthesis of care from the cultural perspective in women with physiological pregnancy from the line of argument. The thematic categories are: a) pregnancy: a natural phenomenon in the woman’s life; b) spirituality and family support: bond tie with God, the family, and the unborn child; c) the midwife: a symbol of traditional practices during pregnancy; d) Positive and negative feelings: search for emotional balance during pregnancy; e) physical exercise: a way preparing for the delivery moment; f) comfort, rest, and general care: actions aimed at caring for the body of the pregnant woman; g) feeding: a way of preserving the wellbeing of the mother and her unborn child; h) avoid consuming non-beneficial substances: provide protection to the unborn child; i) intrauterine stimulation: strengthens the mother-child affective bond; j) heat and cold: equilibrium in the woman’s body; k) sexuality during pregnancy: interpretations by the woman; l) traditional beliefs and myths: a different way of caring herself during pregnancy (Figure 2).

Care from the cultural perspective of the pregnant woman, as construct generated in this research proposes that *pregnancy is conceived as a natural phenomenon in the woman’s life and the family*, is part of the social and biological dimension and from the woman’s vision it develops as an event that requires behaviors and care practices to favor the evolution of the pregnancy, prepare for the delivery, and safeguard the wellbeing of the unborn child.(12,14,22,25,27) In these practices, it is important to indicate the articulation among the categories of *spirituality and family support: bond tie with God, the family, and the unborn child; and the midwife: a symbol of traditional practices during pregnancy.* The family,(14,26,28,30,31) the spouse,(25,26,30) women in the family,(3,14,31) the midwife for some cultures(12,14,15,23,28) and the bond with a superior being (3,26,27,28,31) with that related with physical, affective, economic, and spiritual care, which influences positively on the pregnancy.(3,14,23,31)

Regarding family support, it should be noted that the spouse and other family members are particularly interested in the pregnant woman, desiring the woman and the child have a pregnancy in the best possible conditions.(14,25,26,28,30,31) For this, the role of the women in the family is highlighted, especially that of the mother, who as caregiver and, above all as transmitter of beliefs and care practices, has the experience and knowledge to advice women during pregnancy.(3,14,23,31) The representation of the midwife is a symbol of service and of traditional practices that represent the care of the pregnant woman; these are granted recognition, merit, acceptance, and credibility by the women and their families, by being in charge of the control and care for the woman during pregnancy and delivery.(14,15,23,27) 

It is evident that women during pregnancy act by following their beliefs and traditional practices and advice from the family, but finally the results of the pregnancy “are in hands of God”.(3,27,31) From this perspective, spirituality is an internal guide that provides strength, sense, and significance to the life of the pregnant woman and creates spiritual unity with a supreme being in which the family is involved, seeking to entrust Him with the protection and maintenance of the health of the mother and her unborn child.(28,31) Support from the family, from women in the community or midwives, as well as having a spiritual guide during pregnancy favors *the expression of feelings*. Women seek to maintain an emotional balance that leads them to avoid or stay away from ^situations^ that generate negative feelings, like sadness, anger, or distress,(3,14) thus, permitting them to care for their own wellbeing and that of their children with the expression of positive feelings that generate in them tranquility, happiness, and desires to go forth.(3) Just like pregnant women are aware of the need and importance of emotional care, they also develop activities aimed at caring for and preparing their bodies for a quick delivery and without complications to them and the unborn child.(3) Around this goal, care actions are deployed, among them, *physical exercise: a way of preparing for the delivery.* This is a practice that includes everything related with the body of the future mother, like walking, adopting different positions, and performing some bodily movements, that is, activities that can favor the future mother and avoiding others that cause them harm, to protect themselves during pregnancy and prepare for the delivery.(3,14,16,17,19,31)

For pregnant women, *comfort, rest and general care* are practices aimed at caring for their bodies,(3,18,19,23,28,31) which help them to avoid infections, abortions, premature deliveries, risk of falls, mistreatment to the babies and the children being born with physical defects.(18,23,31) In the search for maternal health protection, pregnant women consider *feeding: a way of preserving the wellbeing of the mother and of her unborn child*, and it is achieved through a feeding change and beneficial practices, like eating well.(2,3,11-13,17,19-23,25-28,30,31) The women include in their eating the consumption "soft foods” or “healthy foods”; among them, vegetables, fruits, meat, chicken, fish, milk and its derivates, which are recognized as foods that serve to gain weight, strengthen the body by avoiding the threat of abortion, preventing anemia, ensuring physical strength, minimizing the physiological effects of the pregnancy, maximizing the baby’s health, and preparing for the delivery;(3,18-21,29-31) and restriction of “strong foods " which could be interpreted as harmful, "heavy", capable of causing harm to the body, such as junk food, snacks and industrialized foods, among others, seen as forbidden during pregnancy.(3,20,21,29-31) Around feeding, rituals also exist aimed at guaranteeing in the mother “to have a good delivery”, from consuming foods that “provide strength”, like foods with salt and *bienestarina* (vegetable flour, added with powdered skimmed milk, enriched with vitamins and minerals); food to “open the flesh” (onion); ingesting hot beverages (cinnamon and castor oil) “favor labor contraction pain”;(3) consuming butter in each meal and drinking abundant liquid facilitate expulsing the fetus.(30)

Women consider *avoiding the consumption of non-beneficial substances* as a way of generating protection to themselves and to the unborn child, recognizing that substances, like alcohol, cigarretes, psychoactive substances and medications are harmful to them and to the child because they are associated with negative effects in the short and long term in the health of the infants.(13,14,19,21,22,24)

*Intrauterine stimulation: enhances the mother-child affective bond* by expressing love and affection with the hope of delivering a happy baby, which develops emotionally, mentally, and socially; generating a human being who is more critical, adaptable, and endowed with emotional intelligence; besides giving the mother the opportunity to reflect on the new role she will soon acquire.(3,19,28,31)

*Heat and cold: balance in the woman’s body*. Cold is considered an enemy that must be fought in as much as possible through heat.(3,28) Cold can enter the woman’s body through different ways and become a threat to the mother and the unborn child.(3,23,31) This leads them to developing a series of practices that let them keep their body in equilibrium between cold and heat and which guarantees them, from their traditional knowledge, to remain well during pregnancy, guarantee the health of the fetus and restore the mother’s health after the delivery.(3,14,23) In synthesis, cold behaves as a sensation they do not like, they feel that what their body presents is not good and is unpleasant.(3)

*Sexuality during pregnancy: interpretations by the woman.* For the pregnant woman, the fact of engaging in sexuality, relating with her partner, and being well are cultural domains with which they identify sexuality in this stage of life.(22) However, fear exists upon practicing the sexuality: hurting or causing physical defects in the fetus, precipitating the moment of delivery,(22,23) and the presence of physical changes during pregnancy altering the image and self-concept, so that sexuality is reduced to the enjoyment of a sexual encounter by the couple without affective feelings.(22)

*Traditional beliefs and myths as a different way of caring for themselves in the pregnancy*; during pregnancy, women stick to diverse customs, myths, and beliefs, which are founded, developed, transmitted, and maintained through knowledge and the experience of a social group social and from a family context in which the pregnant woman is immersed.(28) This is how beliefs are reported related with certain restrictions and mobility during a lunar or solar eclipse,(24,25) attending funerals, weddings(26) and cemeteries,(25) as a way of protecting themselves, preventing deformities in the unborn child, and avoiding disease.(28)

## Discussion

The Meta-ethnography permitted, from first-order interpretations (points of view expressed by the participants of the studies) and second-order interpretations (interpretations reported by the authors of the studies), developing a line of argument (third-order interpretation)(32) that exposes in greater depth existing knowledge on care the women carry out from the cultural context during the prenatal stage. The line of argument links 12 thematic categories, one of them highlights how the pregnancy, in spite of being conceived as a *natural phenomenon in the woman’s life,*(12,14,22,25,27) is a process defined by the social, historical, and cultural contexts that demands in her two important components in caring for herself and her unborn child. The first, is to be prepared psychologically, emotionally, and spiritually.(33) In this sense, the following categories are described: *spirituality*(3,26-28,31) and *family support:* (2,3,14,25,26,28,30,31) *a bond tie with God, the family and the unborn child, and the midwife: a symbol of traditional practices during pregnancy.*(12,14,15,23,28) Family support, women from the community, and the connection with a supreme being during the pregnancy, permit in the woman an affective, economic, and spiritual equilibrium,(3,14,23,31) which leads her to stay away from situations that generate *negative feelings,*(3,14) thus, permitting the care of their own emotional wellbeing with the expression of *positive feelings* that generate in them tranquility and happiness during the pregnancy.(3)

The second component corresponds to the physical preparation, which implies the change of behaviors and habits aimed at the health of the mother and that of the unborn child.(33) This process links seven categories, *the practice of physical exercise;* (3,14,16,19,28,31) *comfort, rest, and general care;*(3,18,19,23,28,31) *feeding: a way of preserving the mother’s wellbeing and that of her unborn child;*(3,12,13,17,19,20,21,23,25-28,30,31) *avoiding the intake of non-beneficial substances;* (13,14,19,21,22,24) *intrauterine stimulation: strengthens the mother-child affective bond;*(3,19,28,31) *heat and cold: equilibrium in the woman’s body;*(3,14,23,28,31) *sexuality during pregnancy: interpretations by the woman.*(22,23) The behaviors and care practices mentioned are framed within a strong system of traditional beliefs*, customs and myths*(24-26,28) that constitute a cultural dimension, which persists, is transmitted through tradition and is part of the reality of the pregnant women to care for their own health and that of their unborn children.(28) 

According to the Theory of Culture Care Diversity and Universality, by Madeleine Leininger, the findings of this research evidence how caring for the woman during the prenatal stage, in each country, is a cultural phenomenon with differences and similarities in its meanings, values, beliefs, life styles, and care practices in the pregnant woman and social and family nucleus. From this principle, the goal of the theory of cultural care, according to Leininger, is to offer care synthesized within Universality (shared or similar characteristics of cultural care) and Diversity (differences or variations of cultural care), so that it can be culturally congruent, safe, beneficial, and significant,(4) aimed at the protection of the mother and her unborn child. 

### Limitations of this study.

The meta-ethnography is a qualitative methodology constituted by seven phases which overlap and repeat as the interpretative synthesis advances. However, no guide exists to clearly transmit the methodology, analysis and synthesis of the reinterpretation of the findings, which becomes an important barrier to the base of the scientific evidence that permits evaluating the methodological rigor, credibility, and reliability of the findings of a meta-ethnography. It is possible that all the studies published on caring for pregnant women from the cultural perspective have not been included in this synthesis, although a complete and extensive search was conducted in Spanish, English, and Portuguese in seven databases and the repository at Universidad Nacional de Colombia.

## Conclusion

The line of argument reflects how behaviors and care practices of pregnant women are aimed at guaranteeing their protection and that of their unborn children. These care practices are configured from knowledge, values, beliefs, and customs, that is, the culture in which the women are born, grow up, and develop. Consequently, care from the cultural perspective in women with physiological pregnancy is a construct that must be understood from the meanings, experiences, and cultural context surrounding the woman’s family, social, and spiritual structure during pregnancy. This interpretative synthesis reflects the need to offer culturally congruent care by the health staff in charge of maternal perinatal care to achieve a dual construct between generic knowledge (emic) and professional knowledge (ethical), which permits their planning and effectively applying care actions adapted to the beliefs of health or disease, values and practices of the pregnant woman and her family, and which helps the pregnant woman to maintain or reach full physical, mental, social, and spiritual wellbeing.

## References

[1] Arévalo E (2007). Gestación y prácticas de cuidado. Av. Enferm..

[2] Bernal MC, Muñoz de Rodríguez L, Ruíz de Cárdenas CH (2008). Significado del cuidado de sí y de su hijo por nacer en gestantes desplazadas. Aquíchan.

[3] Giraldo DI (2007). Significados del cuidado en el preparto. Av. Enferm..

[4] Leininger MM, McFarland MR (2006). Culture care diversity and universality. A worldwide nursing theory. Chapter 1. Culture care diversity and universality theory and evolution of the ethnonursing method.

[5] Leininger MM (1995). Transcultural nursing concepts, theories, research & practices. Chapter 2. Transcultural nursing perspectives: basic concepts, principles and cultural care incidents.

[6] Laza C, Cárdenas FJ (2008). Una mirada al cuidado en la gestación desde la enfermería transcultural. Rev. Cubana Enferm.

[7] Ministerio de Salud y Protección Social (2016). Política de atención integral en salud. Bogotá D.C.

[8] Ministerio de Salud y Protección Social (2018). Lineamiento técnico y operativo de la ruta integral de atención en salud materno perinatal. Bogotá D.C.

[9] Noblit G, Hare D (1988). Meta-Ethnography: Synthesizing Qualitative Studies.

[10] National Collaborating Centre for Methods and Tools (2018). Critical Appraisal Skills Programme. CASP Checklist: 10 questions to help you make sense of a Qualitative Research Checklist.

[11] Ahlqvist M, Wirfält E (2000). Beliefs concerning Dietary Practices during Pregnancy and Lactation. Scand. J. Caing Sci.

[12] Flores C (2003). Saber popular y prácticas de embarazo, parto y puerperio en Yahuío Sierra Norte de Oaxaca. Perinatol. Reprod. Hum.

[13] Medina A, Mayca J. (2006). Creencias y costumbres relacionadas con el embarazo, parto y puerperio en comunidades nativas awajun y wampis. Rev. Perú. Med. Exp. Salud Pública.

[14] Argote LA, Vásquez ML (2007). Ante la desesperanza del desplazamiento: Un hijo sano, el mayor anhelo de la mujer gestante. Colomb. Med.

[15] Chávez RE, Arcaya MJ, García G, Surca TC, Contreras MV (2007). Rescatando el autocuidado de la salud durante el embarazo, el parto y al recién nacido: representaciones sociales de mujeres de una comunidad nativa en Perú. Texto-Contexto Enferm.

[16] Suárez DP, Muñoz de Rodríguez L (2008). La condición materna y el ejercicio en la gestación favorecen el bienestar del hijo y el parto. Av. Enferm..

[17] Hernández LM (2008). La gestación: proceso de preparación de la mujer para el nacimiento de su hijo(a).. Av. Enferm..

[18] Grewal SK, Bhagat R, Balneaves LG (2008). Perinatal beliefs and practices of immigrant Punjabi women living in Canada. J Obstet. Gynecol. Neonatal Nurs.

[19] Rátiva N, Ruíz de Cárdenas CH (2009). Si protegemos la vida y la salud durante la gestación, construimos para los dos un futuro saludable. Av. Enferm..

[20] Ribeiro M, Ferreira S (2010). Práticas alimentares na gravidez: um estudo com gestantes e puérperas de um complexo de favelas do Rio de Janeiro. Ciênc. Saúde Coletiva.

[21] Rodríguez I, Bernal MC (2010). La alimentación de la gestante adolescente: el cambio favorable. Av. Enferm..

[22] Guarnizo M, Pardo MP (2011). El significado de la sexualidad durante la gestación. Av. Enferm..

[23] Ramos CP, Muñoz de Rodríguez L (2011). Prácticas culturales de cuidado de gestantes indígenas que viven en el Resguardo Zenú ubicado en la Sabana de Córdoba.

[24] Barragan DI, Ormond KE, Strecker MN, Weil J (2011). Concurrent use of cultural health practices and Western medicine during pregnancy: exploring the Mexican experience in the United States. J. Genet. Couns.

[25] Choudhury N, Ahmed SM (2011). Maternal care practices among the ultra-poor households in rural Bangladesh: a Qualitative exploratory study. BioMed Pregnancy Childbirth.

[26] Wulandari LPL, Whelan AK (2011). Beliefs, attitudes and behaviours of pregnant women in Bali. Midwifery.

[27] Agus Y, Horiuchi S, Porter SE (2012). Rural Indonesia women’s traditional beliefs about antenatal care. BioMed Res. Notes.

[28] Rendón BJ, Ruiz de Cárdenas CH (2012). Significado de las prácticas de cuidado cultural que realizan las gestantes consigo mismas y sus hijos por nacer en el control prenatal.

[29] De-Graft Aikins (2014). Food beliefs and practices during pregnancy in Ghana: implications for maternal health interventions. Health Care Women Int.

[30] Higginbottom GM, Vallianatos H, Forgeron J, Gibbons D, Mamede F, Barolia R (2014). Food choices and practices during pregnancy of immigrant women with high-risk pregnancies in Canada: a pilot study. BMC Pregnancy Childbirth.

[31] Muñoz M, Pardo MP (2016). Significado de las prácticas de cuidado cultural en gestantes adolescentes de Barranquilla. Aquichan.

[32] Atkins S, Lewin S, Smith H, Engel M, Fretheim A, Volmink J (2008). Conducting a meta-ethnography of qualitative literature: Lessons learnt. BMC Medical Research Methodology.

[33] Osorio JH, Carvajal G, Rodríguez M (2017). Preparación para la maternidad durante la gestación: un análisis de concepto. Invest. Educ. Enferm.

